# Successful Refugee Cohabitation With Host Families: A Concept Analysis and Model Development

**DOI:** 10.1177/08980101241273878

**Published:** 2024-08-30

**Authors:** Areej Al-Hamad, Yasin M. Yasin, Kateryna Metersky, Sepali Guruge, Caitlin Gare, Molly Hingorani

**Affiliations:** 7984 Toronto Metropolitan University, Toronto, ON, Canada; 61781 University of Doha for Science and Technology, Doha, Qatar; 7984 Toronto Metropolitan University, Toronto, ON, Canada

**Keywords:** refugees, cohabitation, concept analysis, holistic nursing, conceptual model

## Abstract

**Purpose:** This concept analysis aims to address the gap in knowledge regarding the concept of successful refugee cohabitation with host families. It seeks to develop a conceptual model that integrates holistic nursing and healthcare practices into refugee cohabitation settings to enhance refugee well-being. **Methods:** A comprehensive literature search was conducted using Walker and Avant's methodology for concept analysis. **Findings:** Successful refugee cohabitation with host families concept characterized by peaceful coexistence, power dynamics, hospitality, and cultural tolerance. Antecedents include refugee shelter needs, societal acceptance, humanitarian solidarity, and legal support. Consequences include refugee inclusion and integration, societal cohesion, psychological well-being, and refugee–host acceptance. **Conclusions:** Successful refugee cohabitation is defined as a private hosting arrangement that embodies shared living spaces and peaceful coexistence amid conflict or crises, fostering resilience and support for displaced individuals by local citizens. It combines hospitality, balanced power, and cultural tolerance, driven by refugee needs for shelter and a commitment to successful refugee settlement and integration. This hosting arrangement promotes societal development and cohesion, economic growth, psychological well-being, and foster inclusion and intergroup tolerance. The integration of holistic nursing principles into refugee cohabitation practices can foster more inclusive and healthy communities.

As civil and political unrest forces people to leave their homes, the need for safe and affordable housing becomes a pressing concern, prompting many refugees to embrace cohabitation as a viable solution (Butler, 2004; Czischke & Huisman, 2018; Oliver et al., 2020). Refugee cohabitation can manifest in diverse forms, including scenarios where refugees relocate internationally into organized environments with volunteer local hosts (Merikoski & Nordberg, 2023). This arrangement suggests that many refugees possess resources that allow them to choose among various housing options and are not merely relegated to living in encampments or substandard shared accommodations (Merikoski & Nordberg, 2023). Structured cohabitation settings facilitate cultural integration and provide a more stable living condition compared to more constrained alternatives (Czischke & Huisman, 2018; Oliver et al., 2020).

In times of financial hardships and housing scarcity, refugee cohabitation emerges as a cost-effective solution that benefits both refugee and non-refugee residents. Cohabitation of this sort can foster collaboration between refugees and hosts while enhancing refugee settlement and integration (Oliver et al., 2020). For example, such living environments can provide a safe space for refugees, aid in language acquisition, as well as nurture social connections that offer invaluable support (Czischke & Huisman, 2018; Merikoski & Nordberg, 2023). Cohousing arrangements of this sort can create opportunities for local citizens to benefit from the diversity within the living space to acquire new skills and languages through co-learning and engagement (Czischke & Huisman, 2018; Oliver et al., 2020). This communal living approach not only provides a practical response to the scarcity of resources but also offers a unique opportunity for refugees to share their cultures and coping strategies, and navigate the intricacies of resettlement (Janssen & Harchaoui, 2019; Katz, 2001).

Emerging from the philosophical backgrounds of humanism and holism (Jasemi et al., 2017), holistic nursing and healthcare emphasize creating environments that promote physical, mental, and social well-being (Dossey & Keegan, 2013; Frisch & Rabinowitsch, 2019; Jasemi et al., 2017). Holistic nursing is grounded in the philosophy that healing is most effective when it considers the whole person—physically, emotionally, spiritually, and socially—rather than focusing solely on specific symptoms or illnesses (Frisch & Rabinowitsch, 2019). This approach aligns with principles such as empathy, cultural sensitivity, and a deep understanding of human needs, which are crucial when addressing the complex scenarios faced by refugees (Jasemi et al., 2017). When applied to refugee cohabitation, holistic nursing can transform environments into nurturing spaces that facilitate not only physical recovery but also psychological resilience and social integration. Refugees often arrive with profound trauma, health disparities, and a disrupted sense of community and identity (Dudek et al., 2022).

Holistic nursing in such settings involves creating comprehensive care plans that include trauma-informed care, mental health support, spiritual counseling, and community-building activities. It also emphasizes the importance of cultural competence among healthcare providers, ensuring that they are sensitive to the cultural backgrounds and specific needs of refugees. The shared living spaces of refugee cohabitation practices align with the holistic care principle of caring for the person as a whole, which is particularly important for people experiencing the stressors associated with displacement (Merikoski, 2022). This underscores the need for a comprehensive concept analysis of refugee cohabitation, particularly for nurses who work with culturally diverse populations. By comprehending the nuances of successful refugee cohabitation as a practice of meaningful hosting and housing arrangement for refugees who are temporarily housed in another country/culture with host families in a planned setting, nursing professionals can develop effective strategies to mitigate the stress associated with cultural adaptation faced by both refugee families and their hosts (Dossey & Keegan, 2013). Gaining insights into refugee cohabitation as a concept can significantly improve nursing practice through the creation of interventions tailored to address the challenges refugee populations encounter in reconciling diverse cultural norms that impact their health and well-being. Additionally, the scarcity of in-depth literature and knowledge on the subject calls for additional development and analysis of the concept. This article intends to (1) analyze the concept of “successful refugee cohabitation with the host families,” and (2) develop a conceptual definition and model for refugee cohabitation that can be integrated as part of holistic nursing and healthcare.

## Method

We employed Walker and Avant's (2019) method for concept analysis (Gunawan et al., 2023). It follows a systematic eight-step process which includes: (1) choosing a concept, (2) establishing the goals or objectives of the analysis, (3) identifying all instances of the concept's use, (4) identifying its defining attributes, (5) finding exemplar model cases, (6) recognizing borderline and contrary cases, (7) determining the antecedents and consequences of the concept, and (8) defining empirical referents (Walker & Avant, 2019). An inductive analysis approach was applied (Elo & Kyngäs, 2008). Model, contrary, and borderline cases are presented below to illustrate refugee cohabitation. They were developed based on the insights from our prior qualitative investigation focusing on the homestay hosting experiences of Ukrainian refugee women in Toronto, Canada (Al-Hamad et al., 2024).

### Identify the Concept and Search Strategy

The Merriam-Webster Dictionary, the American Heritage Dictionary, and the Oxford Dictionary yielded zero results for the term “refugee cohabitation.” While all three sources provided similar definitions, The Merriam-Webster Dictionary offered the most comprehensive definition of “refugee.” Used as a noun, it defines a refugee as “a person who flees to a foreign country or power to escape danger or persecution” (Merriam-Webster, 2024b). In defining “cohabitation” the same method was applied—all three dictionaries were consulted to gather a collection of definitions for the noun “cohabitation.” The Merriam-Webster Dictionary provided the most detailed definition of “cohabitation.” The relevant definitions included: “to live together as or as if a married couple,” “to live together or in company,” and “to exist together” (Merriam-Webster, 2024a).

We then conducted a database search of the academic literature for the terms “refugee” and “cohabitation.” The search strategy was crafted in partnership with a research librarian, and preliminary searches were conducted across multiple databases. This approach, which included all pertinent keywords and index terms, was initially employed to formulate an exhaustive search strategy. The strategy was then modified for other databases to accommodate specific truncations, Boolean operators, and wildcards. Furthermore, the strategy was peer-reviewed by another librarian following the PRESS Peer Review Strategy (McGowan et al., 2016). Additional searches were conducted using Google Scholar and the reference lists of the selected articles were examined to include grey literature like conference proceedings and opinion papers. This method was chosen to guarantee a thorough collection of all pertinent data in alignment with the objectives of this concept analysis, thereby ensuring a comprehensive and exhaustive search and analysis.

**Figure 1.**
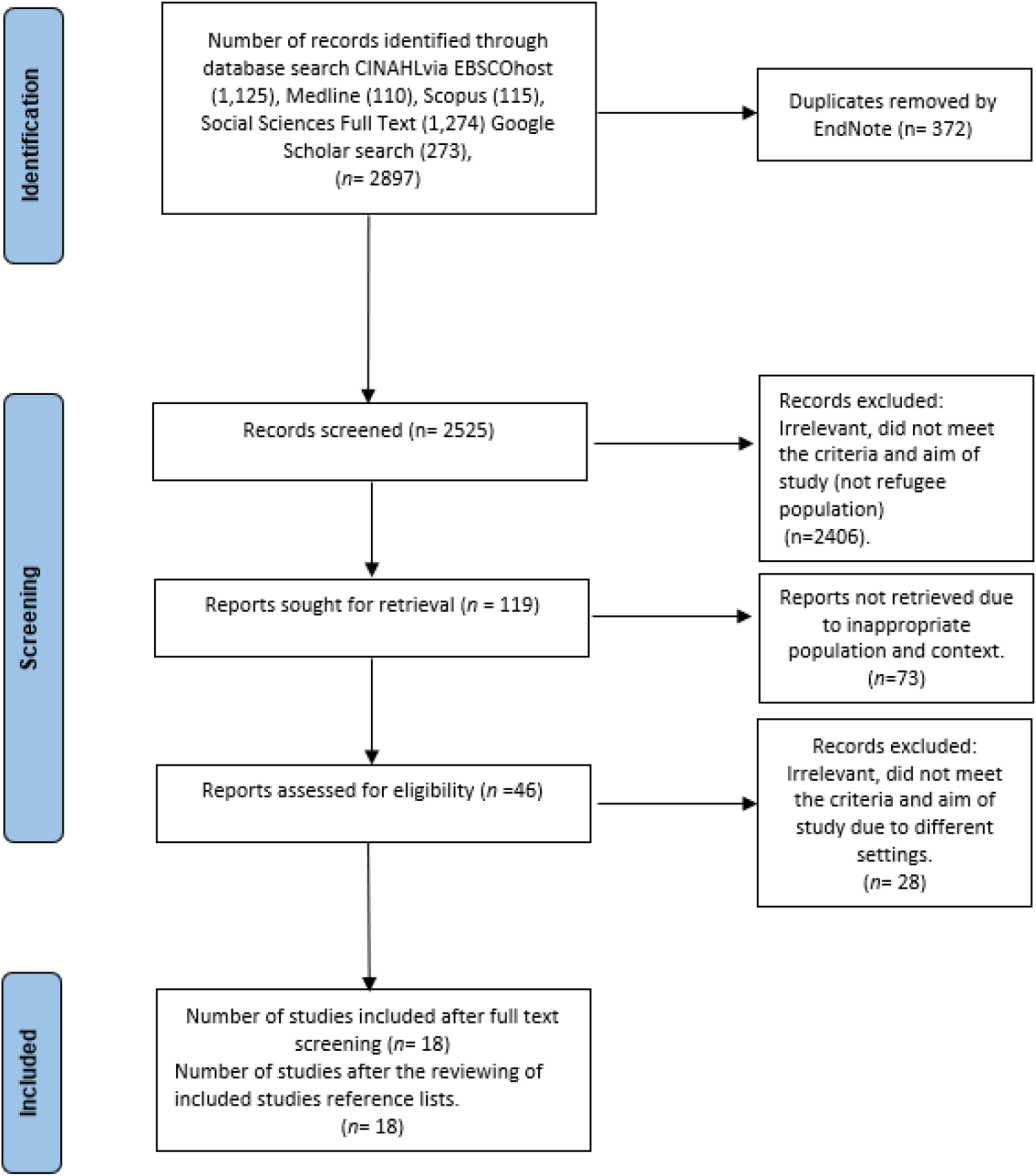
PRISMA flow diagram for selecting articles for inclusion.

A total of 2,897 records were identified through database searches across CINAHL (1,125 records), Social Sciences Full Text (1,274 records), Medline (1,110 records) via EBSCOhost, Scopus (115 records) via Elsevier, and Google Scholar (273 records). Our analysis commenced with an exhaustive review of the existing literature, utilizing search terms including *refugees or asylum seekers or displaced or forced migrants, cohabit, inhabit, habitation, living together, and dwelling* for records published between the years 2000 and 2024 (see Appendix 1 for the search strategy). From these records, duplicates were removed using a reference management software (The EndNote Team, 2013), resulting in 372 records being excluded. Titles and abstracts were screened for relevance by two independent reviewers, AA and CG, with full texts of chosen citations also reviewed by the same individuals. The reasons for excluding papers at the full-text review stage were documented and reported. Disagreements at any stage were settled through discussion or consultation with a third reviewer, YY. The search results and inclusion process were depicted in a PRISMA flow diagram (see Figure 1). Data extraction was conducted by the reviewers, with any disputes resolved similarly. For unclear or missing data, the team contacted authors for additional information. Of the remaining 2,525 records, 2,406 were removed based on their titles and abstracts. Altogether 119 reports were retrieved. Of these, the full text of 46 articles was read and assessed for eligibility. Eighteen studies were deemed eligible and included. The reference lists of these studies were reviewed but no additional studies were found.

## Results

### Relevant Concepts

The studies we examined use a variety of terms interchangeably to refer to the same phenomenon. To comprehensively understand refugee cohabitation concept, our work explored four of the most prevalent concepts explaining phenomena when local citizens host refugee individuals: *cohousing*, *homestay*, *private accommodation/shared living spaces*, and *hospitality*. Although these terms are related, they also carry unique connotations. These terms reflect the historical progression of the refugee cohabitation concept, especially throughout the 20th and 21st centuries. Each concept is discussed below.

#### Refugee Cohousing

Refugee cohousing describes an approach to housing where host citizens (and their families) and refugees coexist within “purpose-built developments or repurposed spaces” designed to cultivate a strong sense of community and mutual support (Czischke & Huisman, 2018). Co-housing involves the clustering of individual household units and communal spaces, with residents dedicated to actively engaging in the planning and administration of the facility (Tummers, 2016). The overarching goal of refugee cohousing initiatives is to promote integration, cultural exchange, and social cohesion by actively encouraging shared responsibilities and communal activities within the living environment (Oliver et al., 2020). These initiatives not only facilitate the physical cohabitation of diverse populations but also serve as catalysts for creating spaces where refugees can learn language skills and develop informal and formal social networks (Mahieu & Van Caudenberg, 2020). Cohousing provides refugees and locals with well-defined personal spaces and roles within shared living environments, effectively fostering a harmonious balance between communal engagement and individual privacy. This approach not only addresses housing challenges but also aids in refugees’ broader integration, self-reliance, and social capital development within host communities (Czischke & Huisman, 2018).

#### Refugee Homestay

Refugee homestay is a multifaceted concept that encompasses the provision of temporary accommodation for refugees within the personal homes of local individuals and families within the host country, offering shelter, safety, and basic amenities until refugees can achieve self-sufficiency or find more permanent housing (Bassoli & Campomori, 2022; Burrell, 2024). The diverse practices of refugee homestay offer personalized support, helping refugees navigate their new surroundings. Host families play an important role in aiding refugees’ adaptation to local customs and daily life, and creating a supportive and family-oriented environment (Bassoli & Luccioni, 2023; Burrell, 2024). Homestay has the potential to offer intimate and authentic insights into local culture, fostering cross-cultural exchange (Bassoli & Luccioni, 2023) through the sharing of customs, traditions, and values, fostering mutual understanding and breaking down cultural barriers. Both refugees and host families can benefit from refugee homestay, leading to more enriched and diverse communities (Boccagni & Giudici, 2022).

However, some authors have highlighted challenges associated with refugee homestays. In homestay practices, refugees are perceived as “guests” who rely on hosts for settlement, thus potentially reinforcing of unhealthy power dynamics that emphasize refugees’ dependence on the generosity and support of hosts (Burrell, 2024). Concerns about refugee independence are heightened when hosting families take over the actions and activities of refugees, potentially hindering their journey toward self-reliance (Ran & Join-Lambert, 2020). This fragile interplay between support and independence is a central consideration in the dynamics of refugee homestay.

#### Refugee Accommodation in Private and Shared Living Spaces

This involves scenarios where refugees live in environments that might be privately owned by citizens who act as hosts (Willems et al., 2020). This may include cases where hosts lease part of their property to refugees or share living spaces with them, enabling refugees to find housing independently of government control. Refugees typically share amenities and responsibilities while living in leased properties or other available and affordable housing options (Dudek et al., 2022; Willems et al., 2020). Such private living spaces allow refugees more control over their living conditions and location, which can significantly improve mental health by providing easier access to resources (Dudek et al., 2022). Moreover, these accommodations enable refugees to customize their living spaces, enhancing a sense of belonging (Willems et al., 2020). Refugees in private residences report greater satisfaction and improved language proficiency than those in shared accommodations, where opportunities to learn the language are often scarce (Baier et al., 2020). Nonetheless, the availability of private accommodation is limited and depends on the readiness of property owners to lease to refugees, who often encounter discrimination and systemic obstacles (Ziersch et al., 2023).

#### Refugee Hospitality

Refugee hospitality has been used in the literature as a multidimensional concept that involves the treatment, reception, and integration of refugees in host societies (Carpi & Pınar Şenoğuz, 2019; Challinor, 2018; Kyriakidou, 2021; McFadyen, 2020). The concept of refugee hospitality extends beyond a simple act of welcoming strangers; it encompasses a complex interplay of power dynamics, cultural perceptions, and media influence within various contexts (Challinor, 2018; Kyriakidou, 2021). The concept of refugee hospitality hinges on an imbalance of power where refugees are labeled as “guests” and a dichotomy is created between guests and the host. This results in situations in which refugees are labeled as “worthy guests” or not based on their adherence to host-imposed rules for guests (Farahani, 2021; Kyriakidou, 2021; McFadyen, 2020). This dynamic positions refugees as recipients of services, aid, and charity, influenced by nationalism, turning hospitality into a power-maintaining tool for the host nation (Carpi & Pınar Şenoğuz, 2019; Challinor, 2018). Cultural values deeply influence refugee hospitality, with cultural identity playing a key role in shaping how refugees are welcomed. However, cultural differences can lead to conflicts, emphasizing the importance and complexity of cultural dimensions in hospitality (Carpi & Pınar Şenoğuz, 2019; Farahani, 2021). Media portrayal of refugees and the sustainability of hospitality practices significantly shape public attitudes toward refugees and perceptions of host societies as either welcoming or not (Carpi & Pınar Şenoğuz, 2019; Kyriakidou, 2021).

### Defining Attributes

Attributes are defined as precise terms that can be used to clarify a concept, aiding in differentiating it from other similar concepts (Walker & Avant, 2019). Our examination of the literature revealed four attributes for the refugee cohabitation concept.

#### Peaceful Coexistence

Peaceful coexistence emerged in the literature we examined as a pivotal attribute in fostering harmonious cohabitation among refugees and local hosts. al-Akhaoui (2008) highlighted the significance of coexistence and civil peace, recognizing the impacts of war and promoting independence by leveraging knowledge in conflict zones to achieve peaceful living. Bell (2021) extended this conversation to the realm of asylum, advocating for an ethos of shared living spaces and responsibility that ensure the dignity and recognition of refugees. Butler (2004) introduced the concept of ethical cohabitation, where emotional and ethical obligations arise from shared spaces and vulnerabilities, emphasizing a cohabitation choice that transcends mere survival. This is complemented by Mahieu and Van Caudenberg (2020), who highlighted the role of meaningful interactions in shared living spaces between refugees and locals in fostering social inclusion and mutual support. Merikoski (2022) delved into the dynamics of home accommodation and the importance of hosting experiences to foster a collective mission and peaceful environment. Similarly, Merikoski and Nordberg (2023) underscored the importance of coexistence, civil peace, and political awareness regarding asylum and refugees in the development of equitable relationships between hosts and refugees. Moreover, Psaltis et al. (2019) advocated for renewed cohabitation based on intergroup contact, coexistence, and interaction, aiming for active engagement and learning from diversity to foster unity and mutual respect.

#### Power Dynamics

Power dynamics significantly influence the cohabitation of refugee populations. Understanding these dynamics involves recognizing their potential to both positively and negatively affect relationships and processes. Constructive power dynamics are essential for fostering peace, empowerment, and mutual respect between refugees and host communities. Such positive dynamics facilitate inclusive decision-making (Bell, 2021), promote dialogue and cultural exchange (Merikoski, 2022; Merikoski & Nordberg, 2023), and enhance conflict resolution training (Butler, 2004). Establishing fair rules that uphold dignity for all is crucial, as it ensures that power supports community cohesion, allowing both refugees and hosts to thrive together (Bell, 2021). However, power can also manifest negatively, potentially exacerbating tensions and reinforcing inequalities. For instance, al-Akhaoui (2008) discusses how societal development and the emancipation of refugee groups require the rejection of internal violence and the effective use of knowledge in conflict regions. Merikoski (2022) and Merikoski and Nordberg (2023) stress the need for a shared understanding of asylum processes and highlight how hosting experiences can be negatively affected by unaddressed power imbalances related to gender, sexuality, class, and cultural identities.

Further studies by Bassoli and Luccioni (2023) delve into the inherent power relations in homestay accommodations, emphasizing the need to mitigate intersectional inequalities to support refugee integration through solidarity and inclusion. Bell (2021) promotes an ethos of responsibility that shifts focus from mere accountability to answerability in decision-making, ensuring community harmony. Gornik (2018) and Luccioni (2023) add to this narrative by advocating for an inclusive environment that supports socio-political stability by understanding and managing the dynamics of interdependence and power, thus addressing both the constructive and destructive potentials of power dynamics in refugee settings. Ott (2016) underscores the importance of moral clarity and shared responsibilities in comprehending the complexities of migration and settlement, emphasizing that an equitable approach to power can significantly improve cohabitation outcomes.

#### Hospitality

Hospitality and homestay practices are pivotal in enhancing refugee cohabitation, emphasizing the creation of inclusive and supportive environments for refugees. Bassoli and Luccioni (2023) delineated the multifaceted nature of refugee homestay, which encompasses hospitality as an inter-individual relationship within the domestic sphere, diverse accommodation arrangements implemented by various groups, and the contentious dimensions of homestay accommodation. Merikoski and Nordberg (2023) extended this discourse by focusing on the accommodation of asylum-seeking migrants in local homes based on hospitality. They highlighted the necessity of recognizing the identity work undertaken by hosts and building strong and equitable relationships between asylum seekers and their hosts. Bassoli and Luccioni (2023) further elaborated on the concept of hospitality, addressing the institutionalization of private hospitality and its capitalization on inequalities and power imbalances. By combining justice with hospitality, Bassoli and Luccioni (2023) argued for the creation of a receptive environment for refugees, where hosting practices are rooted in hospitality and justice, and homestay practices that are thoroughly understood and addressed by the host families. Similarly, Baggio (2020) stressed the inclusion of refugee and minority groups through positive and successful homestay practices to create a social fabric that is rooted in the dynamics of inclusion and hospitality.

#### Cultural Tolerance

Cultural tolerance stands out as an important attribute for fostering positive refugee cohabitation environments. Baggio (2020) discussed the significance of interreligious tolerance and dialogue in fostering multi-ethnic cohabitation and shared communities, illustrating how collaboration between diverse groups can lead to successful integration and creation of a social fabric that is open and respectful of differences. Similarly, Bauman (2018) pointed out the role of cultural heterogeneity and the dialogue surrounding refugee cohabitation can be achieved through education, cultural understanding, and respect. Bassoli and Luccioni (2023) explored how the dynamics of hosting and accommodation intersect with cultural and religious tolerance by providing a practical framework for these concepts. Furthermore, Mahieu and Van Caudenberg (2020) emphasized the importance of urban authorities and local stakeholders in facilitating social integration through the acknowledgment of intercultural identities and experiences, meaningful social interaction between refugees and locals, and the collective communal living that encourages mutual cultural understanding, acceptance, and support. Gornik (2018) explored the ontology of asylum, emphasizing the significance of understanding social, cultural, religious, legal, political, and ethical obligations and the role of cultural and religious understanding in enhancing the asylum seekers’ ability to integrate and participate fully in their host communities. Katz (2001) addressed the positive link between attitudes toward cohabitation and reported cohabitating behavior, noting the influence of incorporating cultural and religious contexts into living arrangements can alleviate the stress and negative experiences associated with refugee cohabitation. Similarly, Ott (2016) emphasized on the need for openness, honesty, and moral clarity in addressing the complexities of cohabitation and highlighted the importance of cultural and religious dialogue in building trust and understanding between refugees and their hosts. Psaltis et al. (2019) discussed the concept of renewed cohabitation and advocate for an environment of tolerance and acceptance, which recognizes and traverses refugee victimization, fostering intergroup cultural and religious dialogue and tolerance and expanded understanding.

### Model Case

A model case is created to ideally represent the concept in practical situations, encompassing all identified attributes from the analysis (Walker & Avant, 2019). The subsequent section presents a typical model case concerning refugee cohabitation in the host country.

Natalia, a Ukrainian refugee, who left the chaos of the Russian invasion, found refuge with the Thompson family in Canada. The Thompsons, who understand the importance of hosting someone from a war-torn country, made their home a welcoming and safe place for Natalia. They offered her a private space while sharing some communal areas, creating a nurturing environment of mutual respect, religious and cultural acceptance, and coexistence. Aware of their privileged position, the Thompsons ensured Natalia felt as an equal and respected, involving her in household decisions and tasks, promoting a balanced and inclusive living arrangement. Their home became a hub of cultural exchange; Natalia introduced them to her Ukrainian heritage including cultural foods and rituals, while the Thompsons shared Canadian traditions, fostering mutual respect and learning. They supported Natalia's integration beyond their home, assisting with legal processes for settlement and connecting her to local communities, embodying the essence of supportive hospitality and homestay practices. The Thompsons recognize the significance of cultural understanding, equality, and community support in making refugees feel truly welcome and valued in their new homes.

Natalia's case with the Thompson family stands as a model case of the refugee cohabitation concept, illustrating how shared living spaces can foster peaceful coexistence, the importance of acknowledging power dynamics and responsibilities, the beauty of supportive hospitality, and the enriching potential of cultural and religious tolerance and dialogue. Through their journey, they demonstrate that when individuals come together under the principles of empathy, respect, and openness, the challenges of displacement can transform into opportunities for unity, growth, and mutual understanding.

### Contrary Case

A contrary case is used to showcase an example that contradicts the model case (Walker & Avant, 2019). The following contrary case provides a divergent view on the concept of refugee cohabitation within the host nation.

Sarah, a Syrian refugee, found temporary shelter with the Anderson family in Canada, escaping conflict from her homeland. However, the Andersons, despite their initial openness to hosting, struggled with the cultural differences, maintaining a strict separation within their home. Sarah was given a small, isolated room, emphasizing a division that hindered any deep connection or sense of belonging. Interactions were minimal and formal, with no effort made to bridge the vast cultural gap or address the power imbalance present. Sarah's presence was barely acknowledged, and she had no say in household matters, feeling sidelined and ignored. The family did not attempt to engage with her culture or facilitate any religious understanding, treating her attempts to share her heritage with indifference or discomfort. This lack of cultural exchange and mutual respect made religious and cultural practices a source of tension rather than an opportunity for learning. The Andersons’ approach to hosting lacked the supportive hospitality essential for integrating refugees into new communities. They focused merely on providing shelter without considering Sarah's broader needs for community integration, legal assistance, or emotional support, effectively overlooking the importance of emotional and social aspects for refugees rebuilding their lives.

In this contrary case, Sarah's placement with the Anderson family illustrates a profound disconnect between the ideals of refugee cohabitation and the reality of her experience. Instead of finding a safe haven where shared living spaces, peaceful coexistence, cultural dialogue, and supportive hospitality flourish, Sarah encountered isolation, misunderstanding, and a lack of genuine engagement, underscoring the challenges and missed opportunities in fostering truly inclusive and supportive environments for refugees.

### Borderline Case

A borderline case illustrates a situation where only some of the concept's attributes are present (Walker & Avant, 2019). Below is an example concerning refugee cohabitation in the context of a host nation.

Fatemeh, an Afghan refugee, was welcomed into Canada by the Wilson family, who offered her a private room in their home, symbolizing an initial step towards hospitality. While they shared meals, deeper communal engagement and cultural integration were minimal, often treating Fatemeh's background as a curiosity rather than embracing it fully into their lives. The Wilsons showed an awareness of their hosting role and the power dynamics involved but rarely incorporated Fatemeh in household decisions, limiting her sense of agency. They provided practical support, like language classes and transportation, aiming to help Fatemeh adjust to her new life. However, their support often lacked the depth needed to foster a genuine sense of belonging and integration, and there was a gap between providing basic needs and offering comprehensive emotional and social support.

In this borderline case, Fatemeh's experience with the Wilson family highlights the complexities of refugee cohabitation. While there were elements of shared living spaces, supportive hospitality, and attempts at cultural dialogue, the full depth and richness of these attributes were not realized. The cohabitation lacked a comprehensive approach to peaceful coexistence, mutual empowerment, and cultural integration, making Fatemeh's transition into her new life in Canada a journey of partial fulfillment amidst missed opportunities for deeper connection and understanding.

### Antecedents

Antecedents refer to events or situations that need to happen before the concept can occur (Walker & Avant, 2019). These antecedents support the phenomenon of refugee cohabitation and highlight the multidimensional needs of refugees, underscore the importance of a holistic approach to aid and policy-making that considers shelter, societal dynamics, humanitarian aid, and legal frameworks to effectively support refugee populations in their new environments. Based on the analysis of the pertinent literature, the antecedents for refugee cohabitation include refugee shelter needs, societal acceptance, humanitarian solidarity, and legal support. Secure shelter is essential for stability (Merikoski & Nordberg, 2023), societal acceptance influences integration and opportunities (Katz, 2001), humanitarian solidarity provides important support and services (Merikoski, 2022), and legal support ensures necessary rights and protections (Butler, 2004). These factors collectively impact refugees’ ability to successfully cohabit and integrate into new communities.

#### Refugee Shelter Needs

Safe and affordable shelter is a fundamental need for all human beings. Ott (2016) highlighted the complexity of refugee shelter needs where accommodation choices are influenced by a variety of factors including safety, access to resources, financial hardship, and the desire for community and belonging. Psaltis et al. (2019) explored the concept of renewed cohabitation, pointing to the need for shelter in facilitating positive integration and the building of social connections that transcended individual victimization. Merikoski and Nordberg (2023) concluded that meeting shelter needs for refugees through intimate and personalized arrangements can contribute to a deeper mutual understanding and respect. Bassoli and Luccioni (2023) highlighted how refugee shelter needs to extend beyond mere physical spaces to encompass considerations of dignity, respect, and equitable treatment. Bell (2021) presented a principle of responsibility that transitions from a focus on accountability to one of answerability regarding the needs of refugees for shelter, emphasizing the preservation of refugee dignity. Additionally, Brekke and Grønningsæter (2017) emphasized three pivotal motives driving refugees toward cohabitation: the separation of refugees from their familial ties, the need for temporary permits as a pathway to securing permanent residency in the host nation, and the necessity of achieving subsistence.

#### Societal Acceptance

Societal acceptance of cohabitation is an important antecedent for refugee cohabitation, influencing how refugees are integrated and accepted within host communities. Katz (2001) pointed to the positive impact of societal attitudes toward cohabitation on actual cohabiting behaviors and successful cohabitation experiences and integration. Bassoli and Luccioni (2023) addressed how the dynamics of hospitality can significantly contribute to societal acceptance of refugee cohabitation. Merikoski and Nordberg (2023) highlighted the significance of host awareness, understanding of refugee experiences, and the development of broader societal discourses around racialized and gendered perspectives of migration on the success of refugee cohabitation. Psaltis et al. (2019) discussed the renewed cohabitation and the variations in acceptance of cohabitation, exploring the interplay between individual victimization, tolerance toward outgroup members, and the critical role of societal acceptance in promoting peaceful coexistence and positive integration of refugees into host communities.

#### Humanitarian Solidarity

The concept of humanitarian solidarity serves as a foundational antecedent for refugee cohabitation, highlighting the importance of ethical conduct, mutual support, and the recognition of shared human values in fostering positive cohabitation environments. Butler (2004) explored the ethics of living together, stressing the need for ethically responsible behavior among all involved by promoting an understanding of ethical duty based on vulnerability, suggesting that exposure to precariousness fosters equality. Bell (2021) introduced an ethos of responsibility that shifts from accountability to answerability in the context of refugee needs, ensuring the dignity of refugees is upheld and emphasizing the significance of recognizing refugees and their human state and needs, promoting a shared, just, and ethical community where refugees live in harmony with their new communities. Gornik (2018) posited that grasping the foundational elements of asylum—which encompass social, cultural, religious, and ethical aspects—is significant and involves delving into the ethical and political dilemmas affecting the state and essence of asylum and their hosts toward cohabitation. Janssen and Harchaoui (2019) determined that the establishment of support networks between individuals fosters unity, enhances life skills, and promotes the development of refugee communities. Merikoski (2022) highlighted collaboration between hosts and refugees in navigating asylum experiences by overcoming previous adversities within a communal living space to secure the right to remain through asylum solidarity and a supportive and ethical framework.

#### Legal Support

Legal support plays a critical role in facilitating refugee cohabitation, offering a foundational structure that supports the integration and peaceful coexistence of refugees within host communities. Butler (2004) emphasizes the importance of ethical cohabitation and the need for policies and legal frameworks that recognize the vulnerability and precarity of refugee situations. Mahieu and Van Caudenberg (2020) highlight the role of societal integration and policies in the success of refugee cohabitation to foster meaningful interactions between refugees and host communities. Bassoli and Luccioni (2023) discuss the need for legal and policy interventions to mitigate power imbalances and inequalities in homestay arrangements and to ensure equitable treatment and integration of refugees. Baggio (2020) underscores the importance of policies that encourage cultural and religious tolerance and dialogue in facilitating cohabitation to create environments where diversity is celebrated. Gornik (2018) points to the necessity for comprehensive asylum policies for refugee cohabitation that addresses the multifaceted nature of refugee experiences, including social, cultural, religious, legal, political, and ethical dimensions.

### Consequences

Consequences refer to the events or situations that may arise following the manifestation of a concept, frequently inspiring fresh perspectives or research paths related to a specific concept (Walker & Avant, 2019). The consequences for refugee cohabitation within host countries include refugee inclusion and integration, social cohesion, psychological well-being, and refugee–host acceptance. These consequences of refugee cohabitation suggest a cyclical relationship between the antecedents and outcomes, where each element influences the other. By examining these consequences, researchers and policymakers can gain valuable insights into the effectiveness of current integration strategies and identify areas needing improvement or further study

#### Refugee Inclusion and Integration

Refugee inclusion and integration is a pivotal outcome that can emerge from effectively managed refugee cohabitation environments. Butler (2004) emphasized how ethical cohabitation underlines the importance of creating living conditions where refugees feel respected and valued for fostering environments where refugees can thrive, leading to successful settlement and integration. Bell (2021) advocated for an ethos of responsibility that shifted focus from accountability to answerability to promote a shared sense of community and responsibility toward refugees, facilitating their inclusion and integration into society. Baggio (2020) and Gornik (2018) emphasized the importance of interreligious tolerance and dialogue in fostering multi-ethnic cohabitation for the successful integration of refugees and to facilitate a community that is open and respectful of differences, enhancing the inclusion of refugees. Mahieu and Van Caudenberg (2020) and Bassoli and Luccioni (2023) underscored the importance of support and solidarity in the successful settlement and integration of refugees through regular, meaningful social interaction between refugees and locals to strengthen social inclusion and mutual support. Brekke and Grønningsæter (2017) emphasized the importance of temporary permits as a pathway to secure permanent residency, self-sufficiency, integration, and eventual settlement within the host country as pivotal for the successful settlement and integration of refugees. Finally, Bauman (2018) underscored the significance of managing refugee migration in a manner that facilitates their successful integration into host communities, directly contributing to effective refugee settlement and assimilation.

#### Social Cohesion

Social cohesion is an outcome of refugee cohabitation that benefits both refugees and host communities. Bauman (2018) emphasized the importance of refugees achieving independence and autonomy through their own initiatives, highlighting the important role of education and respect in attaining social equality and success. Additionally, al-Akhaoui (2008) highlighted the significance of societal development and the emancipation of refugee groups through the rejection of internal violence and the capitalization on the wealth of knowledge in conflict regions to encourage the successful settlement of refugees and their integration. Janssen and Harchaoui (2019) posited the importance of economic opportunities for refugees’ settlement and integration as these opportunities, alongside support mechanisms that help build solidarity and life skills, can contribute to the growth and success of refugee communities. Merikoski and Nordberg (2023) explored how hosting asylum-seeking migrants in local homes over reception centers can cultivate strong, equitable bonds between asylum seekers and their hosts and can significantly contribute to societal growth, unity, and success by enhancing community understanding, inclusivity, and diversity. Ran and Join-Lambert (2020) underlined the role of fostering social connections and a sense of belonging, ensuring the privacy and independence of refugees, and advancing their integration into host societies to contribute to societal development, cohesion, and success by promoting inclusive and adaptable integration processes. Butler (2004) focused on ethical cohabitation practices that respect the dignity and rights of refugees, which can lead to stronger, more cohesive societies that are capable of collective success and development.

#### Psychological Well-Being

Within the context of refugee cohabitation, the psychological well-being of both refugees and hosts emerges as a pivotal consequence. Merikoski and Nordberg (2023) highlighted the intimate nature of cohabitation, interpersonal adjustments both hosts and refugees undergo that fosters mutual understanding and strong relationships, which are essential for the psychological well-being and social integration of refugees. Ott (2016) discussed the unique and diverse experiences of cohabitation, focusing on the honesty and moral clarity needed in navigating the relationships between refugee and host interactions and how these relationships can contribute to a positive mental health environment for both parties. Psaltis et al. (2019) explored the concept of renewed cohabitation, emphasizing the positive integration of internally displaced persons through fostering an environment that recognizes and traverses refugee victimization, the psychological healing and well-being of refugees. Ran and Join-Lambert (2020) emphasized the need to recognize refugees’ distinct experiences, advocating for customized hosting and integration strategies to reduce stress and foster a sense of belonging and autonomy to promote refugees’ mental health in adapting to new settings.

#### Refugee–Host Acceptance

Refugee–host acceptance as a consequence of refugee cohabitation is important for fostering positive relationships and enhancing societal cohesion. Psaltis et al. (2019) investigated the idea of refreshed living arrangements, highlighting the significance of refugee–host acceptance by creating an atmosphere that acknowledges and navigates through personal hardships to aid in the acceptance of refugees. Baggio (2020) underscored the importance of interreligious acceptance and dialogue in promoting multi-ethnic cohabitation and shared community spaces as an approach that not only aids in the successful settlement and integration of refugees but also contributes to the positive refugee–host relationship. Ott (2016) emphasized how resistance and collaboration within the context of cohabitation influence refugee and host interactions and how these interactions that are rooted in openness and honesty can reduce prejudices, building mutual respect between refugees and their hosts. Merikoski and Nordberg (2023) explored hosting asylum seekers in local homes through hospitality, fostering strong and fair connections between refugees and hosts to enhance mutual respect, understanding, and to encourage acceptance. Ran and Join-Lambert (2020) underscored the significance of refugees’ unique experiences to build social connections and inclusion that are important for overcoming social challenges and bolstering acceptance between refugees and hosts, fostering a unified community.

Table 1 outlines the antecedents, attributes, and consequences of refugee cohabitation in host nations.

**Table 1.** Overview of Antecedents, Attributes, and Consequences

Antecedents	Attributes	Consequences
Refugee shelter needs	Peaceful coexistence	Refugees’ inclusion and integration
Societal acceptance	Power dynamics	Societal cohesion
Humanitarian solidarity	Hospitality	Psychological well-being
Legal support	Cultural tolerance	Refugee–host acceptance

## Empirical Referents

The final stage of a concept analysis is the identification of empirical referents, which are measurable occurrences of the phenomenon that facilitate the concept's quantification (Walker & Avant, 2019). This process is significant for assessing tools and frameworks incorporating the concept. Despite the absence of validated scales for evaluating refugee cohabitation in diverse host countries, there are existing scales that were designed to investigate some aspects of refugee cohabitation. For instance, the hospitality aspect of refugee cohabitation was assessed through the Blain and Lashley (2014) scale for hospitableness, utilizing a five-point Likert scale ranging from 1 (strongly disagree) to 5 (strongly agree). Alrawadieh et al. (2023) measured attitudes toward refugees at home through nine items that captured two aspects: warmth (e.g., friendliness, warmth, trustworthiness, tolerance, and sincerity) and competence (e.g., capability, efficiency, organization, and skill), based on Knappert et al. (2021) scale for personal contact and welcoming of refugees that was inspired by the stereotype content model of Fiske et al. (2018). Advocacy intentions were measured by the Wu and Chang (2019) scale. Lastly, Alrawadieh et al. (2023) evaluated the satisfaction derived from hosting refugees by a modified scale developed by Oliver (2014). Upon reviewing the current instrumentation, it becomes evident that while some tools are well-established, there remains a gap in measures that capture the full complexity of refugee cohabitation, particularly in diverse contexts. This discrepancy suggests a need for the development of more nuanced instruments and frameworks that can better accommodate varying cultural, social, and individual factors. Therefore, we recommend further research into the creation of comprehensive and adaptable measurement tools and frameworks, aimed at providing more accurate and context-sensitive evaluations.

## Discussion

We adopted Rast et al. (2024) multidimensional analysis for the Dunn (2002) framework to explore the relationship between cohabitation conditions and refugee health. This approach helped us to conceptually define and develop a model for refugee cohabitation and its connection to holistic nursing. The multidimensional analysis by Rast et al. (2024) informs our deeper analysis of the intersection between cohabitation practices and health among refugees, highlighting the importance of considering a wide range of factors—from physical and biochemical conditions to social and spatial dynamics—in addressing health disparities and promoting integration and well-being in this vulnerable population. Based on our intersectional analysis including the identification of refugee cohabitation antecedents, attributes, and consequences, we developed a conceptual definition of refugee cohabitation.

### Conceptual Definition

Successful refugee cohabitation is conceptually defined as *a private hosting arrangement that embodies shared living spaces and peaceful coexistence amid conflict or crises, fostering resilience and support for displaced individuals by local citizens. It combines hospitality, balanced power, and cultural tolerance, driven by refugee needs for shelter and a commitment to successful refugee settlement and integration. This hosting arrangement promotes societal development and cohesion, economic growth, psychological well-being, and foster inclusion and intergroup tolerance*.

### Conceptual Model Development: Linking Refugee Cohabitation to Holistic Nursing Care

Holistic nursing and healthcare recognize the significance of addressing social determinants of health and fostering supportive environments for refugees to flourish (Dossey & Keegan, 2013; Jasemi et al., 2017). Within the context of holistic nursing and healthcare, investigating refugee cohabitation entails recognizing the intricate interplay of physical, psychological, social, and environmental factors (Bassoli & Luccioni, 2023; Bauman, 2018; Gornik, 2018). Our conceptual model integrates holistic nursing care principles and refugee cohabitation. This model aims to illustrate how holistic nursing practices can address the complex needs of cohabiting refugees, fostering environments that promote their health, well-being, and successful integration. In developing the conceptual model that weaves together the attributes, antecedents, and consequences of the refugee cohabitation concept with the principles of holistic nursing and health, we begin by acknowledging the foundational antecedents that set the stage for refugee cohabitation. These antecedents form the bedrock of a compassionate society and resonate deeply with the ethos of holistic nursing, which prioritizes holism, harmony, healing cultural competence, peace, supportive environments, therapeutic presence, and ethical care (Cohen & Boni, 2018; Dossey & Keegan, 2013). Central to this concept analysis are the defining attributes of refugee cohabitation that mirror the intricate nurse–patient relationships and highlight the importance of understanding power dynamics, healing through empowerment, social integration, and the diverse cultural and psychosocial needs within healthcare settings (Vaismoradi et al., 2022). Just as holistic nursing advocates for care that encompasses the physical, mental, emotional, spiritual, and social dimensions of health, these attributes emphasize the necessity of a nurturing environment for healing and well-being (Dossey & Keegan, 2013).

The consequences of refugee cohabitation parallel the outcomes sought through holistic nursing practices such as offering compassionate, empowering, tailored, and trauma- and violence-informed healthcare services for refugees (Willey et al., 2022). These consequences not only underscore the impact of supportive and inclusive care on refugee and community health but also highlight the interconnectedness of health outcomes. Linking the discussion to holistic nursing, interventions might extend beyond traditional healthcare settings to include advocating for refugees’ culturally competent care, supporting social integration, facilitating community health initiatives, and promoting therapeutic environments conducive to psychological well-being. This linkage underscores the synergetic relationship between the concept of refugee cohabitation and holistic nursing. It illustrates how the principles of holistic nursing—care that is comprehensive, inclusive, and mindful of the complex interplay between various aspects of health—can be applied to address the multifaceted needs of cohabitating refugees and host communities. Through this lens, holistic nursing emerges not just as a healthcare approach but as a vital framework for fostering refugees’ health, well-being, and harmony in dynamic societal contexts. The developed model serves to visually synthesize how the process of refugee cohabitation, through its foundational antecedents, core attributes, and resulting impacts (consequences), aligns with the goals and practices of holistic nursing to promote overall health and well-being.

**Figure 2.**
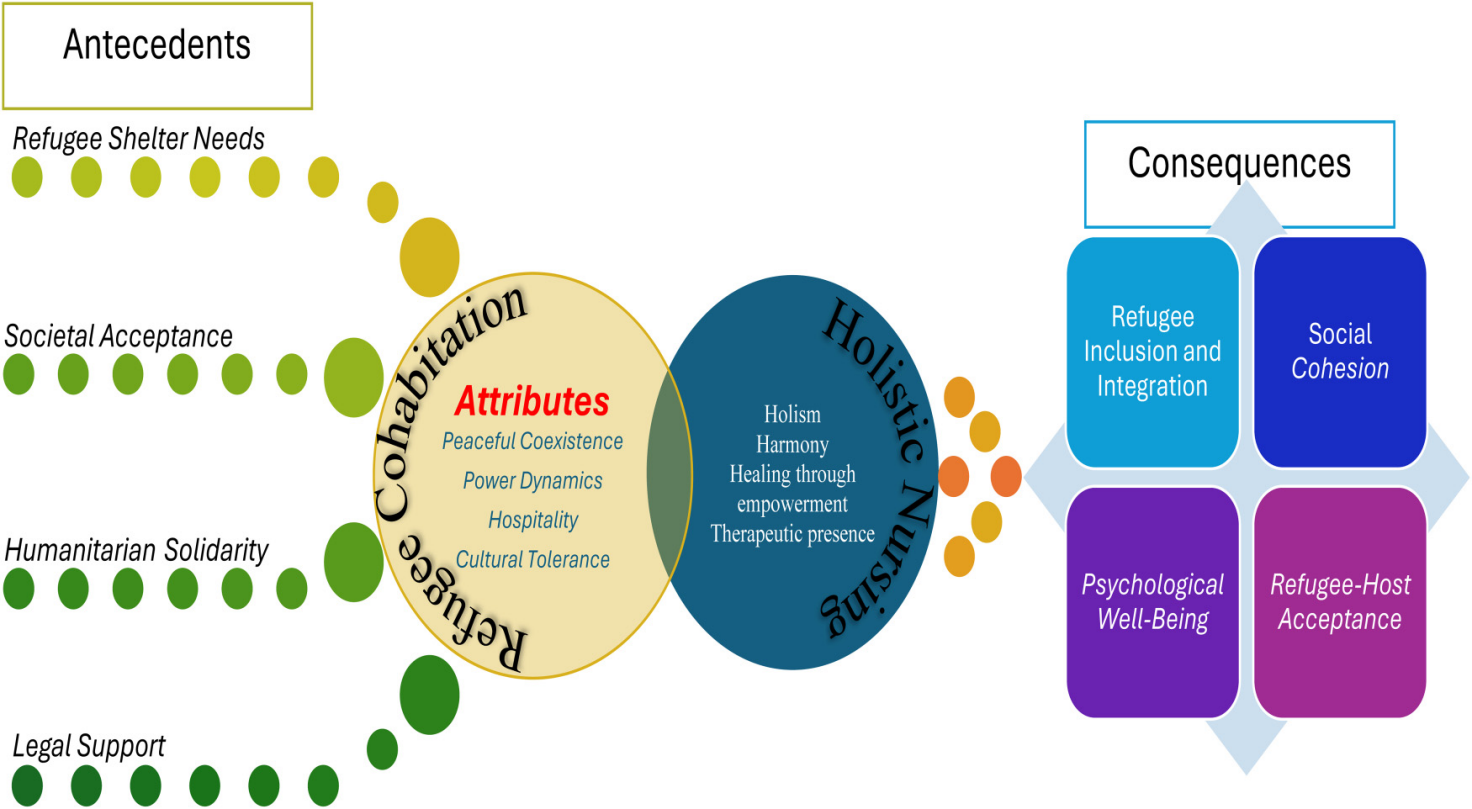
Successful refugee cohabitation and holistic nursing care.

The conceptual model depicted in Figure 2 provides a comprehensive framework for understanding successful refugee cohabitation and holistic nursing care. It organizes the elements into three main components: antecedents, attributes, and consequences. At the core of the model are the attributes essential to successful cohabitation, which include peaceful coexistence, emphasizing harmony among individuals; power dynamics, which addresses the balance of power within cohabitation settings; hospitality, highlighting the importance of a warm welcome for refugees; and cultural tolerance, which stresses the recognition and respect for cultural differences.

Holistic nursing care is portrayed as a pivotal element that mediates the process, emphasizing principles such as holism, which views patient care as an integrated whole; harmony, which promotes balance and peace; healing through empowerment, focusing on empowering patients as a part of the healing process; and therapeutic presence, representing the impactful presence of caregivers in the care setting.

The consequences of implementing these attributes and holistic care practices are manifold. They lead to refugee inclusion and integration, facilitating full participation of refugees within the community; social cohesion, which fosters enhanced community harmony; psychological well-being, improving mental health and emotional stability; and refugee–host acceptance, fostering mutual respect and understanding between refugees and host communities. Overall, this conceptual model serves as a guide for deploying strategies that not only promote successful cohabitation but also enhance holistic care practices, ultimately aiming to improve community integration and health outcomes for refugees (see Figure 2 for the developed model).

### Implications

The concept of refugee cohabitation offers valuable perspectives for enhancing holistic nursing care, which can inform health and social policies, and shape future practices in refugee hosting. Refugee cohabitation not only significantly impacts refugees but also has profound implications for host families and host countries. By acknowledging the mutual impacts on refugees and host families, and leveraging these insights, stakeholders can develop more effective, compassionate approaches to refugee care and integration.

As the global community grapples with the ongoing refugee crisis, understanding the dynamics of cohabitation is essential for developing more effective and sustainable solutions (Gornik, 2018). This calls forth the necessity of action from international stakeholders, academics, and allies (Merikoski, 2022). By acknowledging the complexities inherent in these shared living spaces, policymakers, humanitarian organizations, and host communities can work together to create environments that foster dignity, inclusivity, and hope for those rebuilding their lives in the wake of displacement (Brekke & Grønningsæter, 2017). Understanding the dynamics of refugee cohabitation can lead to more empathetic, culturally sensitive approaches in holistic nursing care and healthcare practices, ensuring the well-being of both refugees and their hosts. Recognizing these impacts, healthcare professionals can offer guidance and support tailored to both refugees and host families, addressing mental, emotional, and social health needs. Moreover, the concept of refugee cohabitation can inform public health policies and healthcare practices to better accommodate the needs of refugees and host communities. This includes developing training programs for healthcare providers in cultural competency and creating health services that are accessible and responsive to the diverse needs of refugee populations. Such initiatives can improve healthcare delivery, reduce barriers to access, and promote health equity. Policies inspired by the principles of cohabitation can encourage a more inclusive society by fostering environments where refugees and host communities can thrive together. Our findings can inform future models for refugee hosting and cohabitation to create frameworks that emphasize cultural exchange, mutual support, and integration. By incorporating the needs and experiences of both refugees and hosts into policymaking and program design, host countries can build more resilient, cohesive communities that are better equipped to welcome and support displaced individuals. Future studies should examine how various host country contexts and readiness levels affect refugee cohabitation and how research findings can inform policies and practices for better support and outcomes in refugee hosting.

### Limitations

We recognize that the concept of refugee cohabitation is complex and affected by various factors, including the socio-economic and cultural backdrop of the host country, and the host's characteristics and readiness to accommodate refugees. Our focus was on understanding the use and diverse interpretations of refugee cohabitation rather than evaluating research quality. Given the dynamic nature of this field and its interdisciplinary scope, our analysis may not capture all facets of the concept, offering a chance for further research to explore the contextual influences on refugee cohabitation more thoroughly.

## Conclusion

Our work delved into the complexities of refugee cohabitation concept, employing the Walker and Avant (2019) framework to unravel its attributes, antecedents, consequences, and implications within host communities. Our concept analysis revealed the nuanced dynamics between refugees and host families, emphasizing the potential for mutual enrichment and cultural exchange that is rooted in coexistence and mutual understanding. Our findings highlight the significance of creating safe and supportive cohabitation environments that benefit refugees, hosts, and hosting countries. Our concept's evolving nature calls for further research, including policies and refugee hosting models that are rooted in best practices in refugee cohabitation and community integration to improve refugee–host relations and well-being and foster a more inclusive society. This work contributes to the discourse on refugee cohabitation and urges a collaborative approach to better understand and support these interactions, enhancing the well-being of refugees and host communities worldwide. In essence, integrating holistic nursing into refugee cohabitation strategies means designing health interventions that are informed by an understanding of the multifaceted human experience. This approach not only aids in healing and adjusting but also empowers refugees to rebuild their lives with dignity and hope in their new communities. By recognizing the intricate challenges present in these communal living areas, policymakers, humanitarian groups, and the communities that welcome newcomers can collaborate to establish settings that promote respect, acceptance, and optimism for individuals seeking to reconstruct their lives after being displaced.

## Appendix 1: Search strategies

### CINAHL via EBSCOhost search strategies:

**Table:**

#	Query	Results
S3	(S1 AND S2)	1,125
S2	refugees or asylum seekers or displaced or forced migrants	18,906
S1	cohabit* OR inhabit OR habitation OR “living together” OR dwelling	286,676

### MEDLINE search strategies:

**Table:**

#	Query	
S3	S1 AND S2	110
S2	refugees or asylum seekers or displaced or forced migrants	59,412
S1	cohabit* OR inhabit OR habitation OR “living together” OR dwelling	65,842
